# Does mental exertion alter maximal muscle activation?

**DOI:** 10.3389/fnhum.2014.00755

**Published:** 2014-09-26

**Authors:** Vianney Rozand, Benjamin Pageaux, Samuele M. Marcora, Charalambos Papaxanthis, Romuald Lepers

**Affiliations:** ^1^Institut National de la Santé et de la Recherche Médicale U1093, Faculty of Sport Sciences, University of BurgundyDijon, France; ^2^Endurance Research Group, School of Sport and Exercise Sciences, University of Kent at MedwayChatham Maritime, UK

**Keywords:** Stroop task, mental fatigue, neuromuscular fatigue, knee extensors, motivation, central fatigue

## Abstract

Mental exertion is known to impair endurance performance, but its effects on neuromuscular function remain unclear. The purpose of this study was to test the hypothesis that mental exertion reduces torque and muscle activation during intermittent maximal voluntary contractions of the knee extensors. Ten subjects performed in a randomized order three separate mental exertion conditions lasting 27 min each: (i) high mental exertion (incongruent Stroop task), (ii) moderate mental exertion (congruent Stroop task), (iii) low mental exertion (watching a movie). In each condition, mental exertion was combined with 10 intermittent maximal voluntary contractions of the knee extensor muscles (one maximal voluntary contraction every 3 min). Neuromuscular function was assessed using electrical nerve stimulation. Maximal voluntary torque, maximal muscle activation and other neuromuscular parameters were similar across mental exertion conditions and did not change over time. These findings suggest that mental exertion does not affect neuromuscular function during intermittent maximal voluntary contractions of the knee extensors.

## Introduction

Mental exertion refers to the engagement with a demanding cognitive task. When performed simultaneously to physical exertion, mental exertion is known to impair endurance performance (Yoon et al., 2009; Mehta and Agnew, 2012). Interestingly, mental exertion also has a negative impact on endurance performance when performed prior to physical exertion (Bray et al., 2008; Marcora et al., 2009; Pageaux et al., 2013). Therefore, there seems to be a clear link between mental exertion and endurance performance in humans.

Contrary to studies on endurance performance, few studies investigated the effect of mental exertion on neuromuscular function. Bray et al. (2008), using a handgrip squeezing task, did not find any decrease in maximal voluntary contraction (MVC) force of the handgrip muscles following 3 min 40 s of mental exertion (incongruent Stroop task). Furthermore, mental exertion induced by 20-min of motor imagery did not alter maximal force production capacity of the elbow flexors (Rozand et al., 2014). Even with 90 min of prior mental exertion induced by the AX-CP test, Pageaux et al. (2013) demonstrated that prolonged mental exertion does not induce a decrease in MVC torque of the knee extensor muscles. Together, these results suggest that mental exertion does not alter maximal force production. However, Bray et al. (2012) measured a significant decrease in force during intermittent handgrip MVCs interspaced with 3-min bouts of high mental exertion (incongruent Stroop task) for a total of 22 min (Bray et al., 2012). In fact, the authors found a significant reduction in maximal force production during the last MVC of this experimental protocol. These results suggest a possible interaction between intermittent MVCs and high mental exertion on maximal force production.

Bray et al. (2012) suggested that the decrease in maximal force production observed during repeated MVCs in the high mental exertion condition (incongruent Stroop task) was caused by a central mechanism, specifically the expenditure of central nervous system (CNS) resources. These authors considered that there is a brain-based energy resource that governs performance of tasks requiring cognitive, emotional, and physical effort regulation (Baumeister et al., 1994; Gailliot et al., 2007). Indeed, it has been proposed that self-regulatory tasks draw upon a common pool of resources that, when depleted, could impair performance during a subsequent physical or mental task requiring self-regulation (Hagger et al., 2010). According to Bray et al. (2012), this expenditure of CNS resources might cause central fatigue, i.e., a decrease in maximal muscle activation during intermittent MVCs, leading to a decrease in handgrip force. However, the late decrease in handgrip force observed in this study occurred without changes in EMG amplitude during the intermittent MVCs. This finding suggests that central fatigue was not induced by this combination of intermittent MVCs and high mental exertion. Unfortunately, EMG amplitude during MVCs is not the most accurate measure of maximal muscle activation (Millet and Lepers, 2004). Therefore, further investigations are necessary to understand the central mechanisms underlying the decrease in maximal force production observed during intermittent MVCs performed in conditions of high mental exertion.

In the present study, we measured the capacity of the CNS to maximally drive the working muscles using the twitch interpolation technique following the guidelines provided by Gandevia (2001). This technique is considered as the gold-standard to assess maximal muscle activation in humans (Gandevia et al., 2013), and consists of delivering a superimposed stimulation (electrical or magnetic) during a MVC to recruit the motor units not voluntarily recruited. If the subject is not able to fully recruit the working muscles, then an additional force will be produced by the stimulation.

In his review on central fatigue, Gandevia (2001) provided guidelines to ensure that subjects exert a true maximal effort during MVCs. Because submaximal effort due to poor motivation can negatively affect measures of maximal muscle activation (Enoka, 1995), these methodological considerations are crucial to ensure the validity of studies investigating the effects of mental exertion on neuromuscular function. Among these methodological considerations, of particular interest is the use of visual feedback performance to maximize voluntary effort (Gandevia, 2001). Unfortunately, in Bray et al. (2012), performance feedback was not available to participants during MVCs. Therefore, it cannot be excluded that the late decrease in handgrip force observed in this study was due to a decrease in motivation to exert a true maximal effort rather than central fatigue. In the present study, visual feedback was provided to the participants for each MVC.

Furthermore, there is evidences that performing the Stroop task involves activation of the trapezius muscle (Waersted and Westgaard, 1996; MacDonell and Keir, 2005) and the forearm muscles (Laursen et al., 2002) are also used in handgrip squeezing. This muscle activity during the Stroop task could be due to holding the sheets (paper-based Stroop task), or selecting the correct responses with a keyboard or a mouse (computer-based Stroop task, Laursen et al., 2002). The continuous use of the upper limb muscles during mental exertion could impact performance during a subsequent physical task involving the same muscle group. Therefore, it cannot be excluded that fatigue within the handgrip muscles (peripheral fatigue, i.e., fatigue produced by changes at or distal to the neuromuscular junction; Gandevia, 2001) contributed to the late decrease in maximal force production observed by Bray et al. (2012). To avoid the potential confounding effect of peripheral fatigue induced by the Stroop task on maximal force production, assessment of a muscle group not involved in the Stroop task (e.g., knee extensor muscles) would be more appropriate.

In this context, the aim of the present study was to analyse the effects of mental exertion on neuromuscular function of the knee extensor muscles. As both mental (Lorist and Tops, 2003; Gailliot, 2008) and physical (Davis et al., 2003; Matsui et al., 2011) exertion has been associated with a reduction in brain glycogen and an increase in brain adenosine which inhibits excitatory activity (Lovatt et al., 2012), we hypothesized that mental exertion would reduce maximal force production during intermittent MVCs. Specifically, we expected that this reduction would be associated with a decrease in maximal muscle activation. To test this hypothesis, we used the twitch interpolation technique to assess maximal muscle activation during intermittent MVCs of the knee extensors following the guidelines of Gandevia (2001) to ensure a true maximal effort.

## Methods

### Subjects

Ten healthy active male subjects (age = 24.5 ± 1.4 yrs, weight = 73.4 ± 1.8 kg, height: 178.1 ± 1.6 cm), volunteered to participate in this study. None of the subjects had any known mental or somatic disorder and written consent was obtained from each subject prior to the study. Experimental protocol and procedures were approved by the local Ethics Committee of the Faculty of Sport Sciences, University of Burgundy in Dijon. All subjects were given written instructions describing all procedures related to the study but were naive of its aims and hypotheses. At the end of the last session, subjects were debriefed and asked not to discuss the real aims of the study with other participants. All procedures were conducted according to the Declaration of Helsinki.

### Procedures

**Figure 1:**
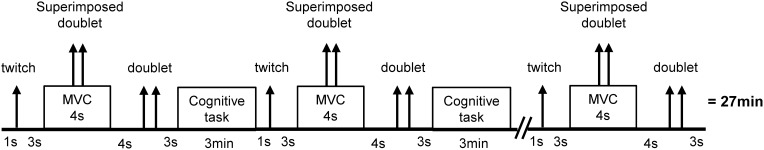
**Overview of the experimental protocol**. The cognitive task was either an incongruent Stroop task (high mental exertion task), a congruent Stroop task (moderate mental exertion task), or watching a movie (low mental exertion, control task). Arrows represent transcutaneous electrical stimuli on the femoral nerve. Single stimuli are represented by one arrow. Double stimuli (100 Hz) are represented by two arrows. Timing was similar between conditions.

Subjects visited the laboratory on four different occasions. During the first visit, subjects were familiarized with the laboratory and the experimental procedures. During the next three visits, subjects randomly performed a mental exertion task lasting 27 min: an incongruent Stroop task, a congruent Stroop task or watching a movie (see *Cognitive Tasks* for more details). Every 3 min, subjects stopped the mental exertion for 15 s to perform neuromuscular tests on the knee extensor muscles. Ten neuromuscular tests were performed for each condition (T0, 3, 6, 9, 12, 15, 18, 21, 24, and 27 min). Motivation was measured before the first neuromuscular test and mood was measured before the first and after the last neuromuscular test. Subjective workload was measured after the final neuromuscular test. For more details see *Psychological Measurements*. An overall view of the protocol can be found Figure 1.

Each subject completed all four visits over a period of 4 weeks with a minimum of 48 h recovery period between visits. All subjects were given instructions to sleep for at least 7 h, refrain from the consumption of alcohol, and not to practice vigorous physical activity the day before each visit. Subjects were also instructed not to consume caffeine and nicotine at least 3 h before testing, and were asked to declare if they had taken any medication or had any acute illness, injury or infection.

### Cognitive tasks

Subjects had to perform a high mental exertion task (incongruent Stroop task involving sustained attention and response inhibition), a moderate mental exertion task (congruent Stroop task not involving the response inhibition process), and a low mental exertion task (watching a movie) for a prolonged period of time (27 min).

#### High mental exertion task

A modified incongruent version of the Stroop-word task (100% incongruent) was used for the high mental exertion condition. Subjects read aloud, as fast as possible, a list of printed words selected in a randomized way (Wallace and Baumeister, 2002). The ink color in which the words were printed was mismatched (e.g., the word “green” printed in blue ink). Participants had to say aloud the color of the ink in which the word was printed (e.g., for the word “green” printed in blue ink, they had to say “blue”). Moreover, for words appearing in red ink, participants were asked to ignore the previous instructions, and say the name of the printed word (e.g., for the word “yellow” printed in red ink, they should say “yellow”). An experimenter recorded the number of incorrect answer with a control sheet, and asked to restart the current line when the answer was incorrect. The modified incongruent Stroop task has been used in several studies on the effects of mental exertion on subsequent physical or mental tasks (Wallace and Baumeister, 2002; Bray et al., 2008; Martin Ginis and Bray, 2010).

#### Moderate mental exertion task

A congruent version of the Stroop-word task was used for the moderate mental exertion condition. Subjects were asked to read aloud, without constraint of speed, a list of printed words. The words and the ink in which they were printed were identical (e.g., the word “green” printed in green ink).

#### Low mental exertion task

The low mental exertion task (control task) consisted in watching a wildlife documentary (“Earth”, directed by A. Fothergill and A. Linfield, 2007). This movie was previously shown to be emotionally neutral (Pageaux et al., 2013).

During all three tasks, subjects were sitting on the ergometer chair used for the intermittent MVCs of the knee extensors. During both incongruent and congruent Stroop tasks, word-sheets were hold by the subjects on their lap, whilst the movie was shown on a computer screen placed on a table in front of them. During the tasks, heart rate was recorded every 5 s during the entire protocol (MVCs and cognitive task) via a heart rate monitor chest strap (Polar RS400, Polar Electro Oy, Kempele, Finland) affixed to the skin near the midpoint of the participant's sternum. Average heart rate was calculated as a psychophysiological index of mental workload (Richter et al., 2008; Yoon et al., 2009).

### Neuromuscular function tests

#### Mechanical recordings

Subjects were seated upright and performed isometric contractions of the right knee extensor muscles. Isometric torque was recorded using a Biodex dynamometer (Biodex Medical System Inc., New York, USA). Two crossover shoulder harnesses and a belt cross above the abdomen limited extraneous movements of the upper body. Neuromuscular tests were performed with the right leg at a knee joint angle of 90° of flexion (0° = knee fully extended) and a hip angle of 90°. The dynamometer axis was aligned with the knee joint axis. Torque signal was digitized on-line at a sampling frequency of 1 kHz using a computer, and stored for analysis with a commercially available software (Acknowledge 4.1.0, Biopac Systems Inc, Goleta, USA). At the beginning of each experiment, subjects performed a standardized warm-up, executing 10 brief submaximal contractions (~50% MVC) of the knee extensor muscles, followed by a 3 min rest (Place et al., 2007). Then participants performed two isometric MVCs to determine their maximal force production. Subjects were motivated to exert maximal effort during MVCs via verbal encouragements provided by an experimenter, and visual feedback corresponding to the torque produced during the previous MVC.

#### Electrical recordings

EMG activity of the vastus lateralis (VL) muscle was recorded with pairs of bipolar silver chloride circular (recording diameter of 10 mm) surface electrodes (Control Graphique Medical, Brie-Comte-Robert, France) positioned lengthwise over the middle of the muscle belly with an interelectrode (center to center) distance of 20 mm. The reference electrode was placed on the opposite patella. Low resistance between the two electrodes (<5 kΩ) was obtained by shaving the skin and dirt were removed from the skin using alcohol. EMG signals were amplified with a bandwidth frequency ranging from 10 to 500 Hz (gain = 500), digitized on-line at a sampling frequency of 2 kHz using a computer, and stored for analysis with commercially available software (Acknowledge, Biopac Systems Inc, Goleta, USA).

#### Evoked contractions

Both single and double (100 Hz frequency) stimulations were used for assessment of neuromuscular function. Transcutaneous electrically-evoked contractions of the knee extensors muscles were induced using a high-voltage (maximal voltage 400 V) constant-current stimulator (model DS7 modified, Digitimer, Hertfordshire, UK). The femoral nerve was stimulated using a monopolar cathode ball electrode (0.5 cm diameter), pressed into the femoral triangle by the same experimenter during all tests. The site of stimulation producing the largest resting twitch and M-wave amplitudes was located and marked on the skin so that it could be repeated reliably through all the protocol. The anode was a large (10 × 5 cm) rectangular electrode (Compex SA, Ecublens, Switzerland) located in the gluteal fold opposite the cathode. The optimal intensity of stimulation (i.e., that which recruited all knee extensor motor units) was considered to be reached when an increase in the stimulation intensity did not induce a further increase in the amplitude of the twitch force and the peak-to-peak amplitude of the VL M-wave (Place et al., 2005). Once the optimal intensity was found, 130% of this intensity was used and kept constant throughout the session for each subject. The supramaximal intensities ranged from 70 to 140 mA. The stimulus duration was 1 ms and the interval of the stimuli in the doublet was 10 ms. Single stimulus was evoked at rest 3 s before the MVCs to investigate the M-wave of the VL muscle associated with the evoked twitch. Paired stimuli were evoked during (superimposed doublet) and 4 s after the MVC (potentiated doublet) to investigate knee extensors muscle contractile properties and to estimate the VAL using the twitch interpolation technique (Merton, 1954). Methodology and supramaximal intensities are according to previous studies (e.g., Place et al., 2007).

#### Data analysis

M-wave peak-to-peak amplitude of the VL muscle was measured during the single stimuli at rest before each MVC. Maximal EMG for the MVC of the VL muscle was quantified as the root mean square (RMS) value over a 0.5 s interval about the same interval of the MVC torque measurement. Maximal EMG RMS values were then normalized to the M-wave amplitude for the respective muscles to obtain the EMG RMS/M-wave ratio. This normalization procedure allows to take into account of the changes in the peripheral parameters (neuromuscular transmission-propagation failure and/or changes in impedance) from the EMG recordings (Place et al., 2007). Potentiated doublet peak (Dt) was measured from the peak torque associated with electrical paired stimuli at rest, 4 s after the end of the MVC. MVC was considered as the peak torque attained during the contraction, and maximal voluntary activation level was quantified by measurement of the superimposed torque response to nerve stimulation during the MVC (Allen et al., 1995; Gandevia, 2001). The voluntary activation level (VAL) was estimated according to the formula:

VAL=(1−superimposed doublet/potentiated doublet) × 100

(MVC _at stimulation_/MVC) corresponding to Strojnik and Komi (1998) correction was used if stimulation was not delivered at the MVC torque value. All VAL calculations were performed for a MVC _at stimulation_ between 95 and 100% MVC in order to ensure reliability of measurement.

### Psychological measurements

#### Motivation

Motivation related to the entire protocol was measured using the success motivation and intrinsic motivation scales developed and validated by (Matthews et al., 2001)[22]. Each scale consists of 7 items (e.g., “I want to succeed on the task” and “I am concerned about not doing as well as I can”) scored on a 5-point scale (0 = not at all, 1 = a little bit, 2 = somewhat, 3 = very much, 4 = extremely). Therefore, total scores for these motivation scales range between 0 and 28.

#### Mood

The Brunel Mood Scale (BRUMS) developed by Terry et al. (2003) was used to quantify current mood (“How do you feel right now?”) before the first and after the final neuromuscular test. This questionnaire contains 24 items (e.g., “angry, uncertain, miserable, tired, nervous, energetic”) divided into six respective subscales: anger, confusion, depression, fatigue, tension, and vigor. The items are answered on a 5-point scale (0 = not at all, 1 = a little, 2 = moderately, 3 = quite a bit, 4 = extremely), and each subscale, with four relevant items, can achieve a raw score in the range of 0 to 16. Only scores for the Fatigue and Vigor subscales were considered in this study.

#### Subjective workload

The National Aeronautics and Space Administration Task Load Index (NASA-TLX) rating scale (Hart and Staveland, 1988) was used to assess subjective workload. The NASA-TLX is composed of six subscales: Mental Demand (How much mental and perceptual activity was required?), Physical Demand (How much physical activity was required?), Temporal Demand (How much time pressure did you feel due to the rate or pace at which the task occurred?), Performance (How much successful do you think you were in accomplishing the goals of the task set by the experimenter?), Effort (How hard did you have to work to accomplish your level of performance?) and Frustration (How much irritating, annoying did you perceive the task?). The participants had to score each of the items on a scale divided into 20 equal intervals anchored by a bipolar descriptor (e.g., High/Low). This score was multiplied by 5, resulting in a final score between 0 and 100 for each of the subscales. Participants completed the NASA-TLX after the entire protocol.

### Statistical analysis

Assumptions of statistical tests such as normal distribution and sphericity of data were checked as appropriate. Greenhouse-Geisser correction to the degrees of freedom was applied when violations to sphericity were present. One-Way repeated ANOVAs were used to compare subjective workload, motivation and average heart rate across the three conditions. A fully repeated 3 × 2 (condition × time) ANOVA was used to test the effects of the entire protocol on mood. Fully repeated 3 × 10 ANOVAs were used to test the effects of condition and time on MVC torque, VAL, EMG RMS/M-wave ratio (VL muscles), peak torque of the doublet and amplitude of the M-wave (VL muscles). Percentage of errors during both Stroop tasks was analyzed with a fully repeated 2 × 9 (condition × time) ANOVA. Significant main effects of time or condition, or interactions were followed up with Bonferonni tests as appropriate.

By using predicted effect size provided by Bray et al. (2012) for changes in MVC torque, an a priori power analysis revealed that nine participants would provide 83 % power to detect differences at an α-level of 0.05. Statistical analyses were conducted using the Statistical Package for the Social Sciences, version 19 for Mac OS X (SPSS Inc., Chicago, IL, USA). A significance level of *p* < 0.05 was used for all analyses. Cohen's effects size f(V) and a priori power analysis were calculated with G^*^Power software (version 3.1.6, Universität Düsseldorf, Germany). Thresholds for small, moderate and large effects were set at 0.2, 0.5, and 0.8, respectively. Data are presented as Mean ± SD in the text and tables, and Mean ± s.e.m. in the figures.

## Results

### Motivation and mood

Intrinsic [*F*_(1.1, 11.1)_ = 1.360, *p* = 0.273, *f*_(*V*)_ = 0.369] and success [*F*_(1.1, 11.1)_ = 1.360, *p* = 0.168, *f*_(*V*)_ = 0.465] motivation related to the entire protocol were similar in all conditions (See Table 1). The mood questionnaire revealed a significant decrease in vigor over time in both conditions [*F*_(1, 9)_ = 7.204, *p* < 0.05, *f*_(*V*)_ = 0.895] with no main effect of condition [*F*_(2, 18)_ = 0.171, *p* = 0.845, *f*_(*V*)_ = 0.139] or condition × time interaction [*F*_(1.276, 11.483)_ = 0.212, *p* = 0.811, *f*_(*V*)_ = 0.153]. The fatigue subscale of the mood questionnaire presented a condition effect [*F*_(2, 18)_ = 5.580, *p* < 0.05, *f*_(*V*)_ = 0.787]. Follow-up tests revealed that subjects rated fatigue lower in the control condition compared to the moderate mental exertion condition (*p* < 0.05). Neither a main effect of time [*F*_(1, 9)_ = 2.748, *p* = 0.132, *f*_(*V*)_ = 0.552] nor a condition x time interaction [*F*_(2, 18)_ = 0.212, *p* = 0.132, *f*_(*V*)_ = 0.503] were found for self-reported fatigue (See Table 1).

**Table 1:** **Motivation and Mood for the three experimental conditions**.

	**Motivation**		**Mood**			
	**Intrinsic**	**Success**	**Fatigue**		**Vigor**	
			**Pre**	**Post**	**Pre**	**Post**
High mental exertion task	18.7 (4.9)	18.9 (5.0)	1.1 (1.3)	2.9 (4.01)	10.6 (1.7)	8.4 (3.0)
Moderate mental exertion task	18.4 (5.0)	18.9 (3.4)	1.1 (1.2)	3.7 (3.4)	10.7 (1.2)	10.1 (1.6)
Control task	17.7 (6.4)	16.3 (4.3)	0.7 (0.9)	1.5 (2.4)	8.6 (2.1)	8.5 (2.8)

Data are presented as means (SD).

### Heart rate, subjective workload and cognitive performance

There was a significant main effect of condition on average heart rate [*F*_(2, 18)_ = 9.396, *p* < 0.01, *f*_(*V*)_ = 1.022]. Follow-up tests showed a significantly lower heart rate during the control task (69.5 ± 5.9 beats/min) compared to during the high mental exertion task (81.7 ± 9.7 beats/min; *p* < 0.05) and the moderate mental exertion task (80.3 ± 8.7 beats/min; *p* < 0.05).

**Figure 2:**
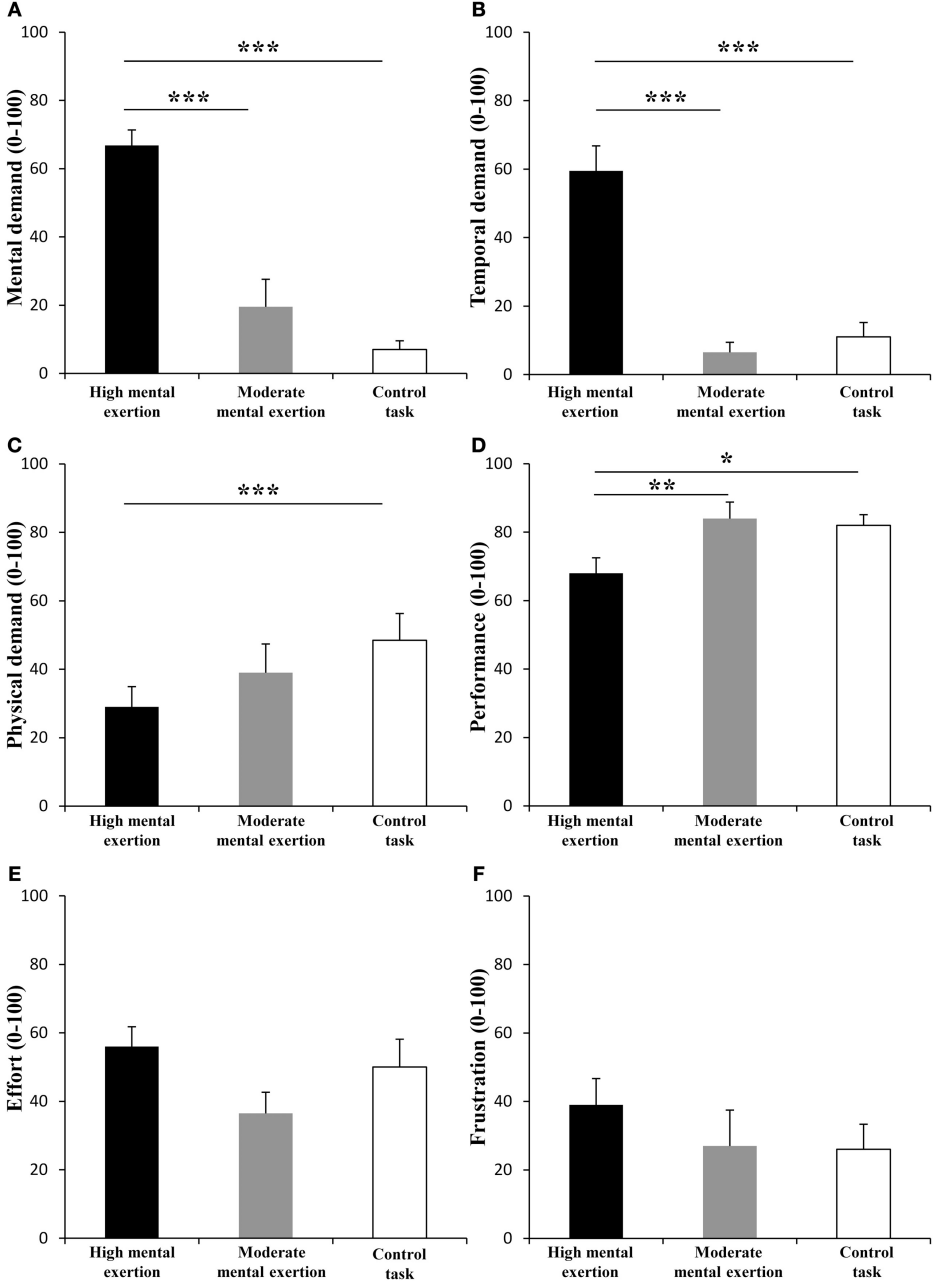
**Effect of mental exertion on subjective workload**. **(A)** Mental demand. **(B)** Temporal demand. **(C)** Physical demand. **(D)** Performance. **(E)** Effort. **(F)** Frustration. ^*^, ^**^ and ^***^: Significantly different (*p* < 0.05, *p* < 0.01 and *p* < 0.001, respectively). Data are represented as means ± s.e.m.

The NASA-TLX scale revealed significant main effects of condition for mental demand [Figure 2A, *F*_(2, 18)_ = 33.061, *p* < 0.001, *f*_(*V*)_ = 1.916], temporal demand [Figure 2B, *F*_(1.2, 11.4)_ = 43.441, *p* < 0.001, *f*_(*V*)_ = 2.194], physical demand [Figure 2C, *F*_(2,18)_ = 4.801, *p* < 0.05, *f*_(*V*)_ = 0.348], performance [Figure 2D, *F*_(2, 18)_ = 6.909, *p* < 0.01, *f*_(*V*)_ = 0.876] and effort [Figure 2E, *F*_(2, 18)_ = 4.428, *p* < 0.05, *f*_(*V*)_ = 0.876]. Follow-up tests showed greater scores for mental and temporal demand after the high mental exertion task than after the moderate mental exertion task (*p* < 0.001) and the control task (*p* < 0.001). In addition, participants rated a higher performance during the moderate mental exertion task (*p* < 0.01) and the control task (*p* < 0.05) compared to during the high mental exertion task. Furthermore, participants perceived higher physical demand during the control task than during the high mental exertion task (*p* < 0.05). Finally, subjects tended to rate a higher effort after the high mental exertion task and the control task than after the moderate exertion task (*p* = 0.064, *p* = 0.088). Frustration (Figure 2F) was not significantly different between the three conditions [*F*_(2, 18)_ = 1.184, *p* = 0.329, *f*_(*V*)_ = 0.362].

Percentage of errors was significantly greater during the high mental exertion task (9.3 ± 4.4 %) than during the moderate mental exertion task (0.5 ± 0.6 %) [main effect of condition *F*_(1, 9)_ = 42.495, *p* < 0.001, *f*_(*V*)_ = 2.171]. However, there was no main effect of time [*F*_(8, 72)_ = 2.570, *p* = 0.093, *f*_(*V*)_ = 0.446] nor a condition × time interaction [*F*_(8, 72)_ = 1.778, *p* = 0.096, *f*_(*V*)_ = 0.444].

### Neuromuscular function

**Figure 3:**
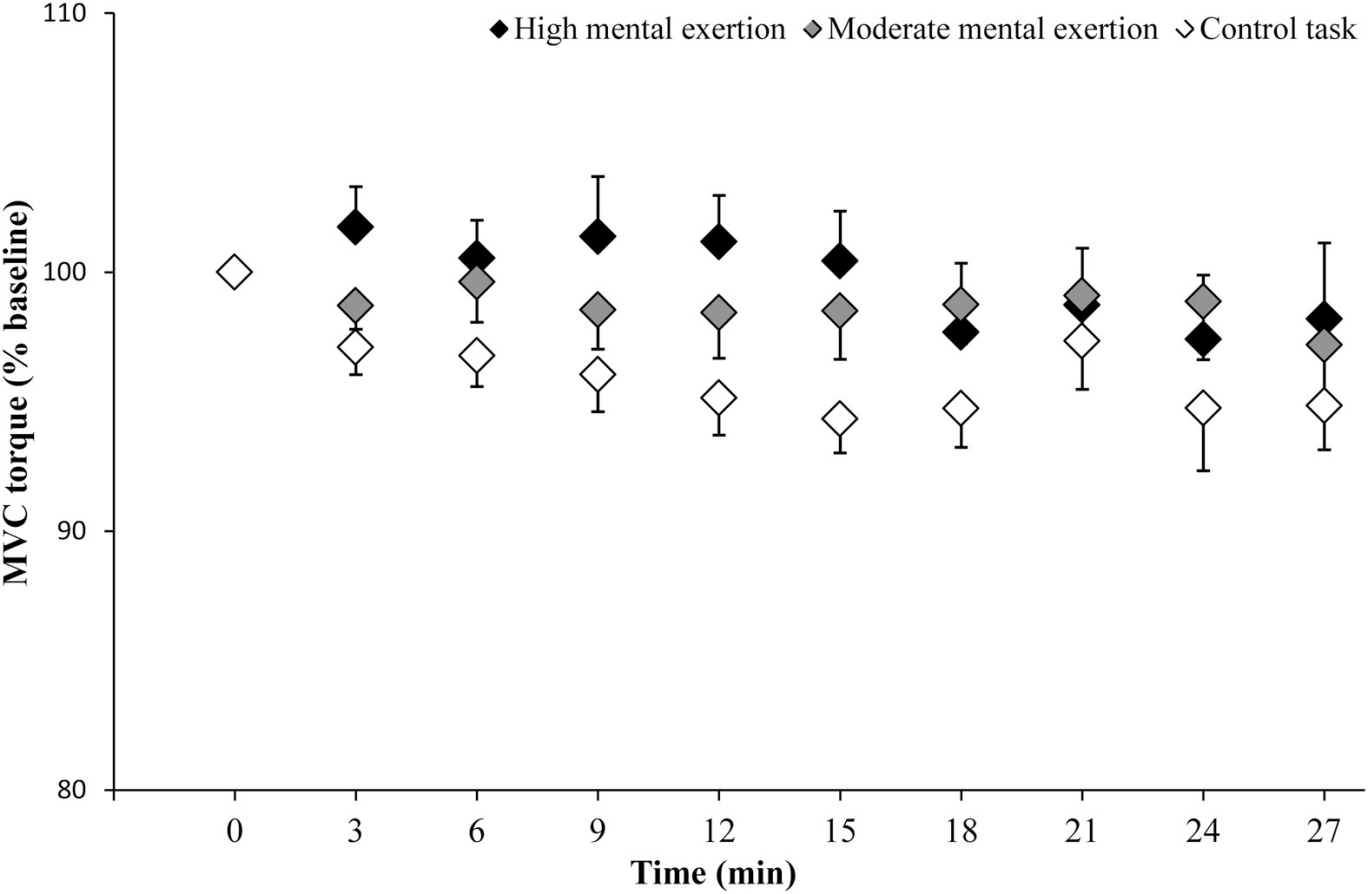
**Maximal voluntary contraction (MVC) torque of the knee extensor muscles during the high mental exertion task (black), the moderate mental exertion task (gray) and the control task (white)**. Data are normalized by the first MVC torque and are represented as means ± s.e.m.

MVC torque of the knee extensor muscles (Figure 3) showed neither a condition × time interaction [*F*_(18, 162)_ = 1.226, *p* = 0.246, *f*_(*V*)_ = 0.369] nor main effects of time [*F*_(2.7, 23.9)_ = 2.088, *p* = 0.13, *f*_(*V*)_ = 0.481] and condition [*F*_(2, 18)_ = 0.293, *p* = 0.749, *f*_(*V*)_ = 0.182].

Maximal muscle activation parameters are shown in Table 2. VAL tended to decrease over time in all three conditions [*F*_(3.7, 42.1)_ = 2.570, *p* = 0.061, *f*_(*V*)_ = 0.534]. However, there was neither a main effect of condition [*F*_(2, 18)_ = 0.121, *p* = 0.887, *f*_(*V*)_ = 0.115] nor a condition x time interaction [*F*_(18, 162)_ = 1.159, *p* = 0.345, *f*_(*V*)_ = 0.359]. Similarly, EMG RMS/M-wave ratio for the VL muscle during the MVCs showed no main effect of condition [*F*_(1.2, 11.2)_ = 1.790, *p* = 0.211, *f*_(*V*)_ = 0.446], no main effect of time [*F*_(3.2, 28.8)_ = 1.728, *p* = 0.181, *f*_(*V*)_ = 0.438], no condition x time interaction [*F*_(18, 162)_ = 1.314, *p* = 0.267, *f*_(*V*)_ = 0.381].

**Table 2:** **Evolution of maximal voluntary contraction (MVC) torque and maximal muscle activation parameters**.

	**Time (min)**									
	**T0**	**3**	**6**	**9**	**12**	**15**	**18**	**21**	**24**	**27**
**MVC (N.m)**										
High mental exertion task	237 (42)	240 (41)	238 (43)	240 (43)	239 (41)	237 (40)	231 (41)	233 (40)	231 (44)	232 (43)
Moderate mental exertion task	239 (33)	236 (35)	237 (30)	235 (33)	235 (33)	234 (31)	235 (33)	237 (37)	236 (38)	232 (37)
Control task	240 (34)	233 (34)	232 (31)	230 (32)	228 (33)	227 (32)	228 (36)	235 (42)	228 (41)	229 (39)
**VAL (%)**										
High mental exertion task	91.2 (5.6)	91.3 (5.6)	89.9 (6.4)	90.6 (7.4)	90.0 (8.8)	88.3 (8.9)	86.4 (9.7)	87.9 (7.9)	88.2 (8.8)	88.3 (8.4)
Moderate mental exertion task	90.9 (6.6)	88.7 (8.7)	90.0 (5.4)	87.4 (6.2)	90.6 (6.6)	88.9 (6.6)	89.3 (7.2)	88.6 (8.0)	87.8 (6.9)	86.9 (6.9)
Control task	90.8 (5.9)	90.1 (6.1)	89.8 (7.3)	88.9 (6.9)	89.7 (6.6)	89.9 (7.0)	89.1 (6.2)	89.3 (4.6)	88.5 (6.0)	89.2 (4.9)
**RMS/M**										
High mental exertion task	0.053 (0.011)	0.056 (0.009)	0.051 (0.012)	0.052 (0.014)	0.051 (0.011)	0.050 (0.013)	0.045 (0.010)	0.050 (0.012)	0.050 (0.011)	0.051 (0.010)
Moderate mental exertion task	0.055 (0.012)	0.054 (0.017)	0.055 (0.016)	0.054 (0.020)	0.049 (0.014)	0.051 (0.011)	0.052 (0.013)	0.051 (0.011)	0.047 (0.010)	0.051 (0.011)
Control task	0.058 (0.008)	0.055 (0.011)	0.054 (0.012)	0.053 (0.013)	0.053 (0.010)	0.055 (0.012)	0.055 (0.010)	0.056 (0.011)	0.053 (0.012)	0.058 (0.015)

Data are presented as means (SD) for MVC torque, voluntary activation level (VAL) and EMG RMS/M-wave ratio of the vastus lateralis muscle.

Peripheral parameters of neuromuscular function are presented Table 3. The amplitude of the potentiated doublet remained constant over time in all three conditions [*F*_(2.5, 22.4)_ = 2.427, *p* = 0.101, *f*_(*V*)_ = 0.519]. There was neither a significant main effect of condition [*F*_(2, 18)_ = 0.306, *p* = 0.740, *f*_(*V*)_ = 0.185] nor a condition x time interaction [*F*_(18, 162)_ = 0.714, *p* = 0.794, *f*_(*V*)_ = 0.280]. Furthermore, M-wave amplitude data for the VL muscle showed no significant main effect of time [*F*_(1.9, 17.1)_ = 2.645, *p* = 0.102, *f*_(*V*)_ = 0.542], no significant main effect of condition [*F*_(1.2, 10.4)_ = 0.991, *p* = 0.356, *f*_(*V*)_ = 0.331] and no condition x time interaction [*F*_(18, 162)_ = 0.706, *p* = 0.802, *f*_(*V*)_ = 0.281].

**Table 3:** **Evolution of peripheral parameters of neuromuscular function**.

	**Time (min)**									
	**T0**	**3**	**6**	**9**	**12**	**15**	**18**	**21**	**24**	**27**
**POTENTIATED DOUBLET (Nm)**										
High mental exertion task	99.8 (17.6)	97.4 (17.9)	96.0 (20.8)	96.1 (19.3)	90.5 (16.4)	93.5 (17.3)	86.6 (18.3)	89.0 (18.1)	91.0 (16.9)	90.2 (15.0)
Moderate mental exertion task	94.7 (21.8)	92.1 (27.6)	93.0 (24.3)	89.7 (25.3)	94.6 (19.2)	91.6 (16.6)	88.5 (15.2)	88.6 (20.1)	89.3 (19.0)	90.6 (17.8)
Control task	95.5 (19.4)	94.2 (20.1)	93.1 (18.8)	93.6 (19.8)	90.9 (21.2)	91.8 (19.5)	90.2 (19.9)	90.9 (19.2)	89.6 (20.0)	89.1 (19.0)
**M-WAVE AMPLITUDE (mV)**										
High mental exertion task	17.9 (1.3)	18.1 (1.4)	18.0 (1.4)	17.9 (1.3)	17.8 (1.5)	17.8 (1.2)	17.7 (1.3)	17.6 (1.1)	17.6 (1.2)	17.6 (1.3)
Moderate mental exertion task	18.8 (2.2)	18.7 (2.8)	18.8 (2.5)	18.7 (2.6)	18.8 (2.6)	18.7 (2.4)	18.7 (2.4)	18.5 (2.5)	18.5 (2.2)	18.4 (2.5)
Control task	18.1 (1.6)	18.3 (1.7)	18.4 (1.6)	18.3 (1.5)	18.2 (1.6)	17.8 (1.5)	17.7 (1.5)	17.5 (1.5)	17.4 (1.6)	17.5 (1.6)

Data are presented as means (SD) for potentiated doublet and M-wave amplitude of the vastus lateralis muscle.

## Discussion

The aim of this study was to test the hypothesis that mental exertion would reduce maximal force production during intermittent MVCs by decreasing maximal muscle activation. Contrary to our hypothesis, neither high mental exertion nor moderate mental exertion altered maximal force production and maximal muscle activation during intermittent MVCs of the knee extensors. These findings demonstrate that the combination of intermittent MVCs and high mental exertion does not reduce the capacity of the CNS to drive to the working muscles.

### Manipulation checks and motivation

Similarly to Bray et al. (2012), the subjects of the present study rated higher mental and temporal demand after the high mental exertion task compared to the moderate mental exertion and the control tasks. Additionally, we found a higher average heart rate during both high and moderate mental exertion tasks compared to the control task. These subjective and psychophysiological manipulation checks suggest that we were successful in inducing different levels of mental exertion across conditions.

Because submaximal effort due to poor motivation can negatively affect measures of maximal force production and maximal muscle activation (Enoka, 1995), we followed Gandevia (2001) six-point guideline to maximize motivation during MVCs: (1) maximal efforts should be accompanied by some instruction and practice, (2) feedback of performance should be given during the MVCs, (3) verbal encouragements should be given, (4) subjects must be allowed to reject efforts that they do not regard as maximal, (5) feedback for repeated MVCs should be updated, and (6) rewards for repeated testing should be considered to ensure consistent motivation between sessions. In our study, points 1–5 were respected, and the point 6 was controlled by completion of a motivation questionnaire at the beginning of each session. All subjects presented similar intrinsic and success motivation to perform the entire protocol across all three conditions. Therefore, we are confident that poor motivation did not negatively affect measures of maximal force production and maximal muscle activation in our study.

### Effects of mental exertion on maximal force production and maximal muscle activation

It is well known that both mental (Lorist and Tops, 2003; Gailliot, 2008) and physical (Davis et al., 2003; Matsui et al., 2011) exertions reduces brain glycogen and increases brain adenosine, a metabolite known to inhibit excitatory activity at neural level (Lovatt et al., 2012). Therefore, our hypothesis was that high mental exertion would induce a significant decrease in maximal force production during intermittent MVCs by reducing the capacity of the CNS to drive the working muscles. Contrary to our hypothesis, we found that high mental exertion did not induce a further decrease in torque and maximal muscle activation during intermittent MVCs of the knee extensors. The present findings support those of Pageaux et al. (2013) and Bray et al. (2008) who showed that short (3 min 40 s of incongruent Stroop task) or prolonged (90 min of the AX-Continuous Performance Task) mental exertion did not alter MVC torque and maximal muscle activation of both upper and lower limbs muscles. Indeed, torque, VAL and EMG RMS/M-wave ratio during MVCs remained constant over time even in the high mental exertion condition. Taking all together, these results suggest that mental exertion does not reduce the capacity of the CNS to drive the working muscles.

The most plausible explanation for the lack of interaction between mental exertion involving response inhibition and maximal muscle activation is that these CNS functions involve different brain areas (Pageaux et al., 2013). Indeed, functional magnetic resonance imaging studies showed that central fatigue during index finger abduction exercise is associated with decrease in activation of the supplementary motor area and to a lesser extent, in parts of the paracentral gyrus, right putamen and in a small cluster of the left parietal operculum (Van Duinen et al., 2007). Interestingly, none of these brain areas is significantly associated with mental exertion involving response inhibition. This cognitive process is significantly associated with activity of the pre-supplementary motor area and the anterior cingulate cortex (Mostofsky and Simmonds, 2008). Therefore, it is neurobiologically plausible that the differentiation in brain areas involved in response inhibition and maximal muscle activation could explain why mental exertion does not reduce the capacity of the CNS to drive the working muscles. However, other neurophysiological techniques have to be used to assess brain activation during both mental and physical exertion (Mauger, 2013). Thus, prefrontal cortex activity could be assessed by near infra-red spectroscopy (Derosière et al., 2013), as its activity increases along with the perception of effort (Berchicci et al., 2013) and the level of physical performance (Mandrick et al., 2013).

### Mental exertion and peripheral fatigue

Contrary to the present study, Bray et al. (2012) observed a significant decrease in maximal force production during intermittent MVCs. In addition to poor motivation exacerbated by high mental exertion, another possible explanation for the results of Bray et al. (2012) is the presence of peripheral fatigue induced by the incongruent Stroop task. Recent studies on the upper limb suggested that mental exertion could induce an earlier onset of peripheral fatigue on shoulder muscles (Mehta and Agnew, 2012). This is supported by an activation of the trapezius muscle when handgrip squeeze is performed in the same time of a shoulder abduction in interaction with mental exertion (MacDonell and Keir, 2005). This activation of the trapezius muscle or forearm muscles (Waersted and Westgaard, 1996; Laursen et al., 2002) could be due to holding the sheets (paper-based Stroop task), or selecting the correct responses with a keyboard or a mouse (computer-based Stroop task).

We avoided these confounding effects of the Stroop task by testing maximal muscle activation of the knee extensor muscles, not functionally connected with muscle affected by combined mental and physical exertion (e.g., shoulder muscles). Indeed, in the present study, both resting evoked contraction and M-wave amplitude remained constant during the 27 min of combined mental exertion and intermittent MVCs of the knee extensors. These results demonstrate that the 3 min intervals between MVCs were sufficient to avoid any exercise-induced peripheral fatigue, and that the Stroop task did not significantly affect the knee extensors neuromuscular function.

Although the results of Bray et al. (2012) may be explained by poor motivation of their subjects and the peripheral fatigue induced by the Stroop task, it has to be acknowledged that the effects of mental exertion on intermittent MVCs could differ between upper and lower limbs. However, this differentiation between upper and lower limbs is unlikely. Indeed, previous studies demonstrated that upper limb (Bray et al., 2008), lower limb (Pageaux et al., 2013) and whole-body endurance performance (Marcora et al., 2009; Pageaux et al., 2014) are impaired by mental exertion.

### Limits and conclusion

Despite a small sample size, the statistical power to test our two main hypotheses (condition × time interactions on MVC torque and VAL) reached an acceptable level of 0.8 (Cohen, 2013). Furthermore, it has to be noticed that the control condition presented the greater decrease in MVC torque (high mental exertion: −2.1%; moderate mental exertion: −2.9%; control condition: −4.6%), suggesting that, if a Type II error was present, the negative effect on neuromuscular function would occur during the control condition and not during the high mental exertion condition. Therefore, it is highly unlikely that we failed to detect a significant effect of mental exertion on the capacity of the CNS to drive the working muscles.

From a psychobiological point of view, the present study suggests that mental exertion does not alter maximal muscle activation. From an applied point of view, these findings combined with previous observations on upper and lower limbs (Bray et al., 2008; Pageaux et al., 2013) indicate that mental exertion does not reduce maximal force production. Therefore, unlike endurance tasks (Marcora et al., 2009; Pageaux et al., 2013), short duration physical tasks requiring high level of force should not be negatively affected by mental exertion.

### Conflict of interest statement

The authors declare that the research was conducted in the absence of any commercial or financial relationships that could be construed as a potential conflict of interest.
